# Transcriptomic characterization and potential marker development of contrasting sugarcane cultivars

**DOI:** 10.1038/s41598-018-19832-x

**Published:** 2018-01-26

**Authors:** Shiqiang Xu, Jihua Wang, Heyang Shang, Youzong Huang, Wei Yao, Baoshan Chen, Muqing Zhang

**Affiliations:** 1 State Key Lab for Conservation and Utilization of Subtropical Agric-Biological Resources, Guangxi University, Nanning, 530005 China; 2 Crop Research Institute of Guangdong Academy of Agricultural Science, Guangzhou, 510640 China

## Abstract

Sugarcane (*Saccharum officinarum* L.) is an important crop for sugar production and bioenergy worldwide. In this study, we performed transcriptome sequencing for six contrasting sugarcane genotypes involved in leaf abscission, tolerance to pokkah boeng disease and drought stress. More than 465 million high-quality reads were generated, which were *de novo* assembled into 93,115 unigenes. Based on a similarity search, 43,526 (46.74%) unigenes were annotated against at least one of the public databases. Functional classification analyses showed that these unigenes are involved in a wide range of metabolic pathways. Comparative transcriptome analysis revealed that many unigenes involved in response to abscisic acid and ethylene were up-regulated in the easy leaf abscission genotype, and unigenes associated with response to jasmonic acid and salicylic acid were up-regulated in response to the pokkah boeng disease in the tolerance genotype. Moreover, unigenes related to peroxidase, antioxidant activity and signal transduction were up-regulated in response to drought stress in the tolerant genotype. Finally, we identified a number of putative markers, including 8,630 simple sequence repeats (SSRs) and 442,152 single-nucleotide polymorphisms (SNPs). Our data will be important resources for future gene discovery, molecular marker development, and genome studies in sugarcane.

## Introduction

Sugarcane produces more than 70% of the sugar worldwide and is also one of the most important crops for biofuels^1^. As an alternative energy source, many countries have implemented plans to produce alcohol from sugarcane^2–4^. China produced 10.556 million tons of sugarcane during the 2014/2015 harvest, which was 2.762 million tons less than in 2013/14. Production reduced to 9.3 million tons in 2015/16, with particularly sharp decreases in Guangxi, where the major constraints in sugar production were the increased labor and production cost, decreased sugar price and plantation area, and the more serious drought stress and disease incidence.

The cost for sugarcane production has quickly risen in China over the past several years, especially with regard to labor costs for manual harvest as well as production costs for over fertilization, pesticides and herbicides. Currently, more than 95% of sugarcane is manually harvested in China, in contrast to several other countries where mechanical harvesting dominates. Removal of dead leaves from cane stalks (defoliation) is a major task during harvesting, which contributes greatly to increased costs. One way to address these problems and facilitate green-cane harvesting is the development of easy-defoliating cultivars^5^. Biotic and abiotic stresses are becoming more serious problems due to the severe singleness of the sugarcane variety in the main sugarcane growing areas, where ROC22 has occupied almost 70% of the total sugarcane area for more than 10 years and more than 80% of sugarcane grown in the upland areas where irrigation is not available^6^. In recent years, the incidence of sugarcane pokkah boeng disease in China showed a trend of gradually increasing, and has become the main disease during the early growth of sugarcane. Therefore, the release and extension of new cultivars with strong resistance to disease and drought and higher ratooning ability is urgently needed for sugarcane production in China.

Modern sugarcane cultivars are derived from the interspecific hybridizations between *S*. *officinarum*, *S*. *spontaneum*, and other species in order to obtain disease resistance, high sucrose content, and high yield^7^. The chromosome number of these modern cultivars ranges from 100 to 130, indicating high levels of polyploidy and aneuploidy^8^. The genome size of sugarcane cultivar R570 was estimated to be approximately 10 Gb, and average monoploid genome sizes of *S*. *officinarum* and *S*. *spontaneum* were estimated to be 985 Mb and 843 Mb, respectively^9,10^. To date, no complete sugarcane genome sequence has been reported, which restricts the development of functional genomics and modern breeding.

Expressed sequence tags (ESTs) provide an important resource for discovering novel genes and assisting in the genome annotation of sugarcane. The first EST database for sugarcane was constructed from leaf roll tissue (meristematic region)^11^. The Brazilian SUCEST project, the largest EST database (about 238,000 ESTs), was carried out to construct a genetic linkage map and to identify cell wall-related genes in 26 cDNA libraries from many tissues and sugarcane cultivars^12–14^. All of the ESTs are deposited in the Sugarcane Gene Index (version 3.0), which contains 282,683 ESTs and 499 complete cDNA sequences, resulting in 121,342 unigenes. However, more than 10,000 sugarcane coding genes have yet to be identified, highlighting the necessity for further studies on the sugarcane transcriptome^15^.

RNA sequencing (RNA-seq) is an effective tool for deciphering the transcriptome and is particularly useful for species lacking a sequenced genome. The large quantity of reads obtained can be assembled for gene annotation, gene discovery, gene expression, and identification of regulation patterns in organisms. RNA-seq has also been used for discovery of putative molecular markers (SNPs, SSRs) to facilitate trait mapping and marker-assisted selection without the requirement for a reference genome^16^. RNA-seq technology has been used for transcriptome analysis in many species, such as rice, maize, soybean, and sugarcane^17–20^.

In this study, the transcriptomic characterization of six contrasting sugarcane genotypes was compared in response to pokkah boeng, drought and leaf abscission. The sequenced data from the Illumina Hiseq. 2500 were *de novo* assembled and annotated against several public databases. The putative markers (SSRs, SNPs) were identified, which will be useful for screening variation among the contrasting sugarcane genotypes.

## Results

### Sequencing and assembly

Each RNA sample was extracted and sequenced using the Illumina paired-end sequencing technology. After quality assessment and data filtering, more than 465 million high-quality reads were used for *de novo* assembly (Supplementary Table S1). The raw reads were deposited in the Sequence Read Archive (SRA) at GenBank databases ID: SRP127762. A total of 471,654 transcripts were assembled with a mean length of 1,450 bp and an N50 length of 2,067 bp using Trinity software (Table 1). These transcripts represented a total of 93,115 unigenes, with a mean length of 910 bp and an N50 of 1,774 bp. The average length and N50 of the assembled unigenes was higher than those observed for *S*. *spontaneum* (801 bp and 1,337 bp) and sugarcane variety GT35 (460 bp and 640 bp) using similar sequencing technologies^21,22^, demonstrating the high quality of our sugarcane transcriptomic sequences. In total, more than 43,308 unigenes (46.51%) were over 500 bp in length, 25,486 (27.37%) over 1,000 bp, and 12,278 (13.19%) longer than 2,000 bp (Table 1).

**Table 1:** Summary of assembled transcripts and unigenes of the sugarcane transcriptome.

Length Range (bp)	Transcript	Percentage	Unigene	Percentage
200–300	45,855	9.72%	27,843	29.90%
300–500	54,346	11.52%	21,964	23.59%
500–1000	100,799	21.37%	17,822	19.14%
1000–2000	150,068	31.82%	13,208	14.18%
>2000	120,586	25.57%	12,278	13.19%
Total Number	471,654	—	93,115	—
Total Length	683,858,120	—	84,779,317	—
N50 Length	2,067	—	1,774	—
Mean Length	1,450	—	910	—

### Functional annotation

**Figure 1:**
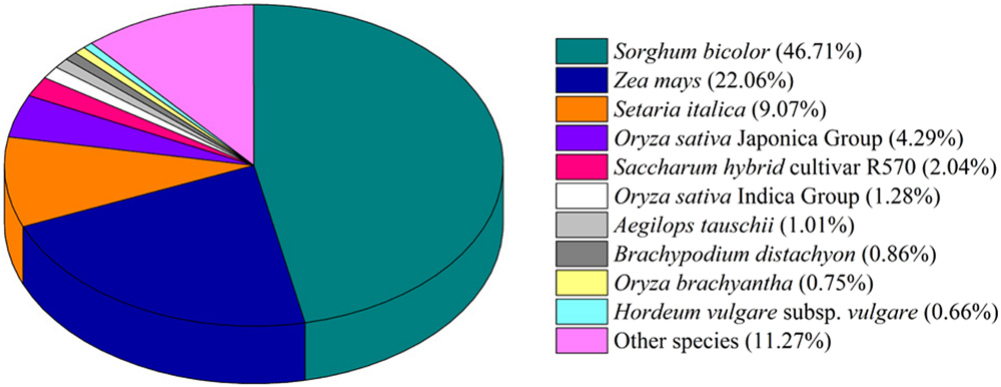
Species distribution of the top BLAST hits against the NR database for the assembled unigene (E-value ≤ 1.0 × 10^−5^). Nearly 46.71% of unigenes showed significant homology with that of *Sorghum bicolor* and 22.06% showed significant similarity with that of *Zea mays*.

The lack of a reference sugarcane genome is a challenge for gene function prediction and utilization of the transcriptome dataset. Overall, 43,526 (46.74%) unigenes were annotated against at least one of the public databases (Table 2 and Supplementary Table S2). A total of 42,042 (45.15%) unigenes showed homologs in the NR database, while 22,660 (24.34%) unigenes had similarity to proteins in the Swiss-Prot database. However, a total of 49,589 (53.26%) unigenes could not be annotated, suggesting that these unannotated unigenes might be novel genes, although some of these unigenes may represent non-coding RNAs. Among the BLASTx top hits, 19,632 (46.71%) were matched to *Sorghum bicolor* proteins, followed by *Zea mays* (9,272; 22.06%), *Setaria italica* (3,812; 9.07%), and *Oryza sativa* Japonica Group (1,804; 4.29%) (Fig. 1). These results were consistent with previous reports due to the higher collinearity in the genic regions between sugarcane and sorghum genomes^23,24^. Interestingly, only 857 (2.04%) unigenes showed significant homology with those of the *Saccharum hybrid* cultivar R570, which was consistent with a previous report^20^. This result might be due to the lack of reference genome sequence and limited public data in sugarcane, and might also point to the high genetic variation among different sugarcane genotypes. A total of 20,738 (81.37%) of the unigenes with sizes over 1,000 bp showed homologous matches, whereas only 7,705 (27.67%) of the unigenes shorter than 300 bp were annotated.

**Table 2:** Functional annotation of assembled unigenes.

Database	Annotated Number	Percentage	300–1000 bp	≥1000 bp
NR	42,042	45.15%	14,329	20,611
Swis-Prot	22,660	24.34%	6,727	13,238
GO	30,677	32.95%	9,751	16,179
Pfam	25,853	27.76%	7,251	15,563
KOG	21,108	22.67%	6,427	11,403
COG	10,575	11.36%	2,742	6,517
KEGG	12,367	13.28%	3,872	6,571
All annotated	43,526	46.74%	15,083	20,738
Total unigenes	93,115	—	—	—

### Cluster of Orthologous Group (COG)

**Figure 2:**
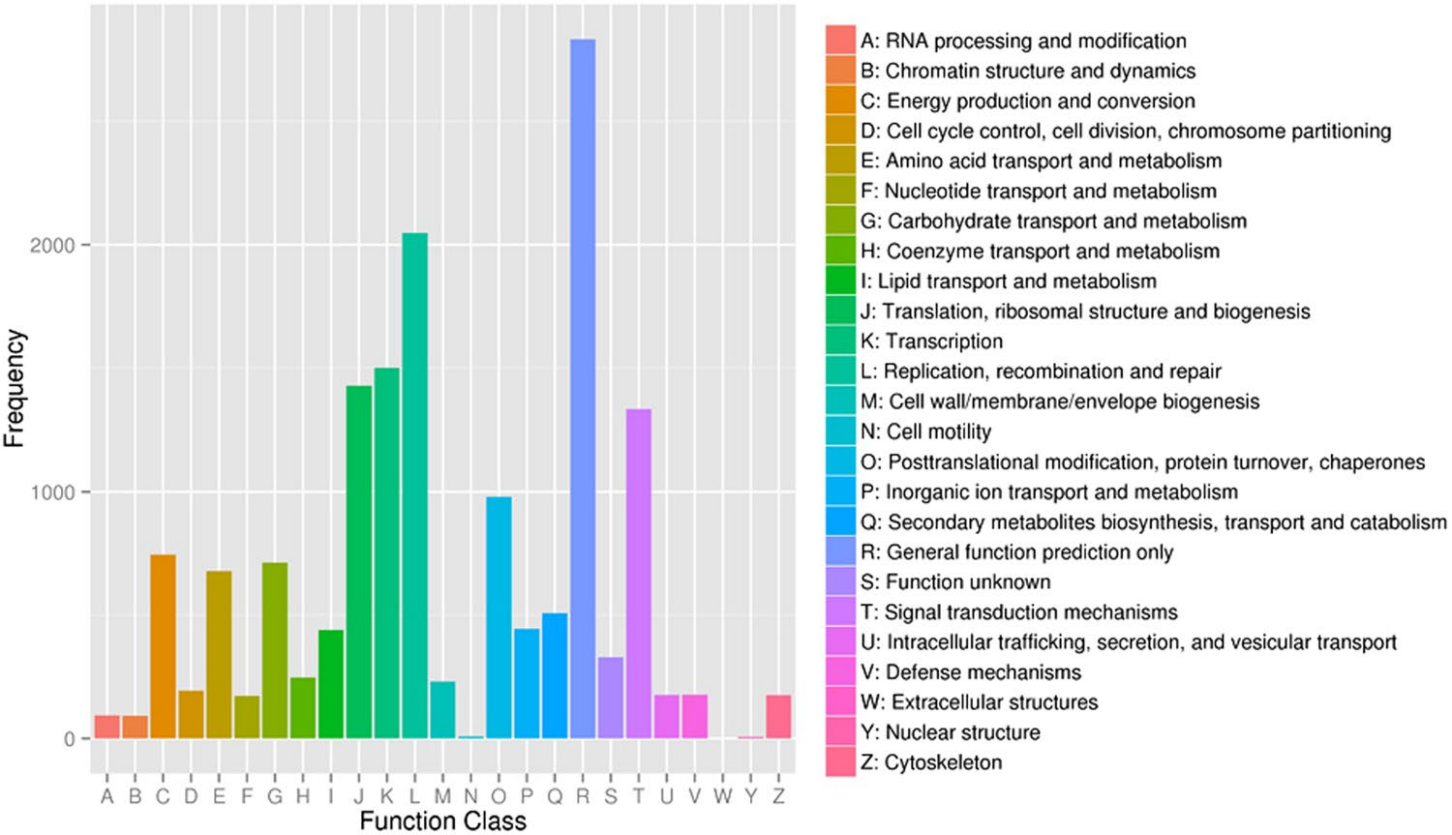
COG classification of sugarcane assembled unigenes with an E-value threshold of 1.0 × 10^−5^ against COG databases. Total 10,575 unigenes were grouped into 25 COG classifications, and the cluster of general function prediction represented the largest group, accounting for 18.22%.

All assembled sugarcane unigenes were searched against the COG database for functional prediction and classification. A total of 10,575 (11.36%) unigenes were assigned and classified into 25 COG categories (Fig. 2). The cluster for ‘general function prediction only’ (2,833; 18.22%) represented the largest group, followed by ‘replication, recombination, and repair’ (2,047; 13.16%), ‘transcription’ (1,500; 9.64%), ‘translation, ribosomal structure and biogenesis’ (1,429; 9.19%) and ‘signal transduction mechanisms’ (1,333; 8.57%). Only a few unigenes were assigned to ‘cell motility’ and ‘nuclear structure’ (8 and 7 unigenes, respectively). In addition, 330 unigenes were assigned to a class representing unknown function. Unigenes in categories representing ‘energy production and conversion’ (745; 4.79%), ‘carbohydrate transport and metabolism’ (712; 4.58%), ‘signal transduction mechanisms’ (1,295; 8.53%), and ‘defense mechanisms’ (178; 1.14%) may be used to develop molecular markers of agronomic traits, such as biomass, sugar content, and abiotic and/or disease resistance.

### Gene Ontology (GO)

**Figure 3:**
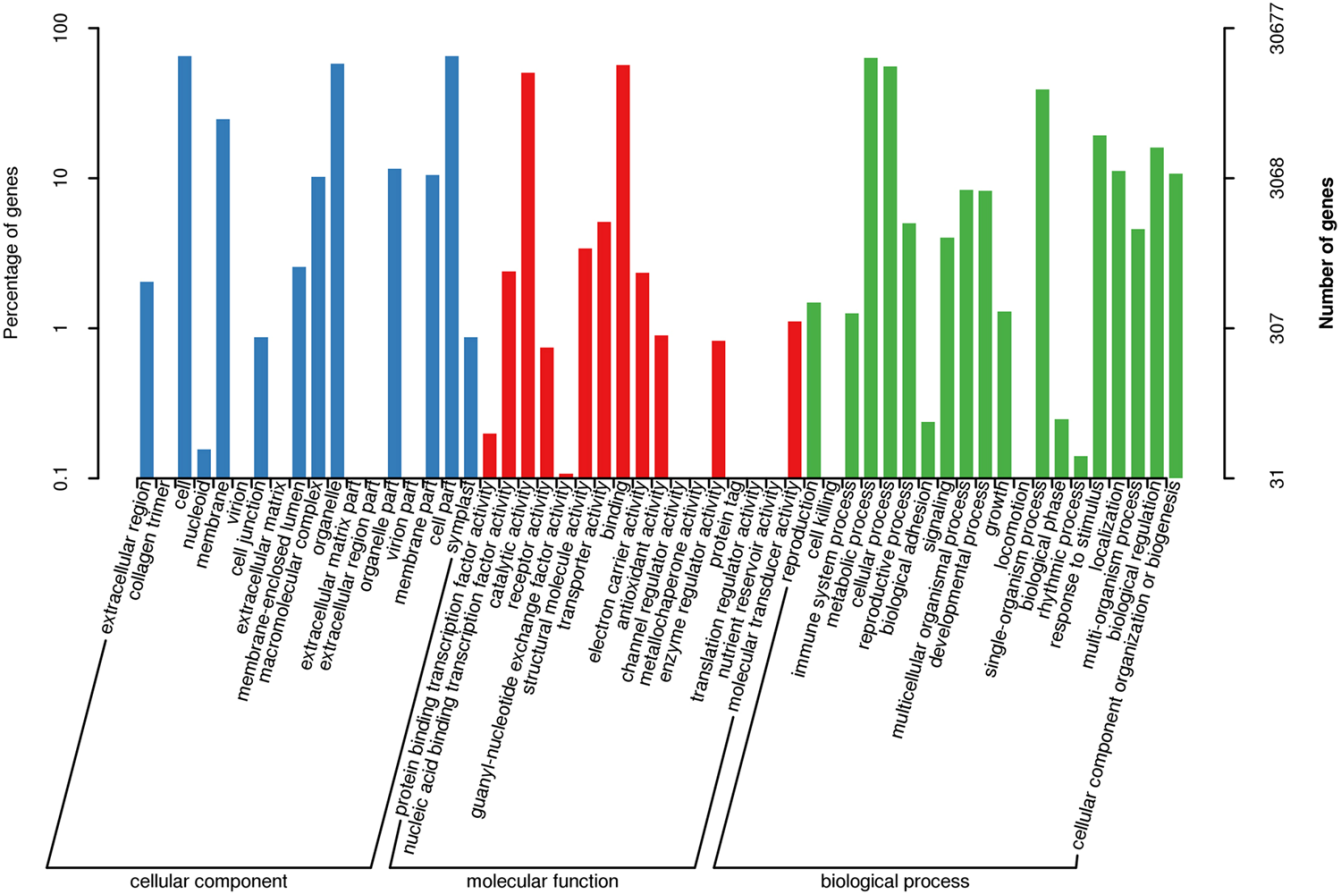
Gene ontology (GO) classification of the assembled unigenes. A total of 30,677 unigenes with BLASTx matches were assigned to three main categories: biological processes (76,711; 39.94%), cellular components (77,191; 40.19%), and molecular functions (38,159; 19.87%).

GO was used to classify the unigene function predictions according to three main categories: molecular function, biological process, and cellular component. A total of 30,677 (32.95%) unigenes were classified into 55 GO functional sub-categories (Fig. 3). Cellular components represented the majority of the functional terms (77,191; 40.19%), followed by biological processes (76,711; 39.94%) and molecular functions (38,159; 19.87%). Within the cellular components category, ‘cell’ and ‘cell part’ (19,953; 10.39%) was the most dominant group, followed by ‘organelle’ (17,728; 9.23%), ‘membrane’ (7,600; 3.96%), and ‘organelle part’ (3,559; 1.85%). Within the molecular function category, ‘binding’ (17,434; 9.08%) and ‘catalytic activity’ (15,430; 8.03%) were prominently represented, and within the biological process category, ‘metabolic process’ (19,385; 10.09%) and ‘cellular process’ (17,026; 8.86%) were the most enriched. The unigenes involved in the categories representing ‘signaling’ (1,235), ‘receptor activity’ (229), ‘response to stimulus’ (5,937), and ‘antioxidant activity’ (276), might be closely related to drought or/and disease response and provided valuable information for further studies.

### KEGG pathway

To further understand the biology and intricate metabolic pathways of sugarcane, all assembled unigenes were annotated using the KEGG pathway database. In total, 12,367 (13.28%) unigenes were annotated to 126 pathways (Supplementary Table S3). The most highly represented pathways were ‘ribosome’ (1,009; 8.16%), followed by ‘carbon metabolism’ (452; 3.66%), ‘biosynthesis of amino acids’ (393; 3.18%) and ‘protein processing in endoplasmic reticulum’ (388; 3.14%). Unigenes in the ‘plant-pathogen interaction’ pathway (358; 2.90%) may be useful for studying the resistance mechanisms to pokkah boeng disease in sugarcane. Pathways associated with leaf abscission and drought stress, such as ‘plant hormone signal transduction’ (276; 2.23%), ‘glutathione metabolism’ (177; 1.43%) and ‘ubiquitin mediated proteolysis’ (189; 1.53%) will be the focus of future studies.

### Comparative Transcriptomic Characterization of Contrasting Sugarcane Cultivars

**Figure 4:**
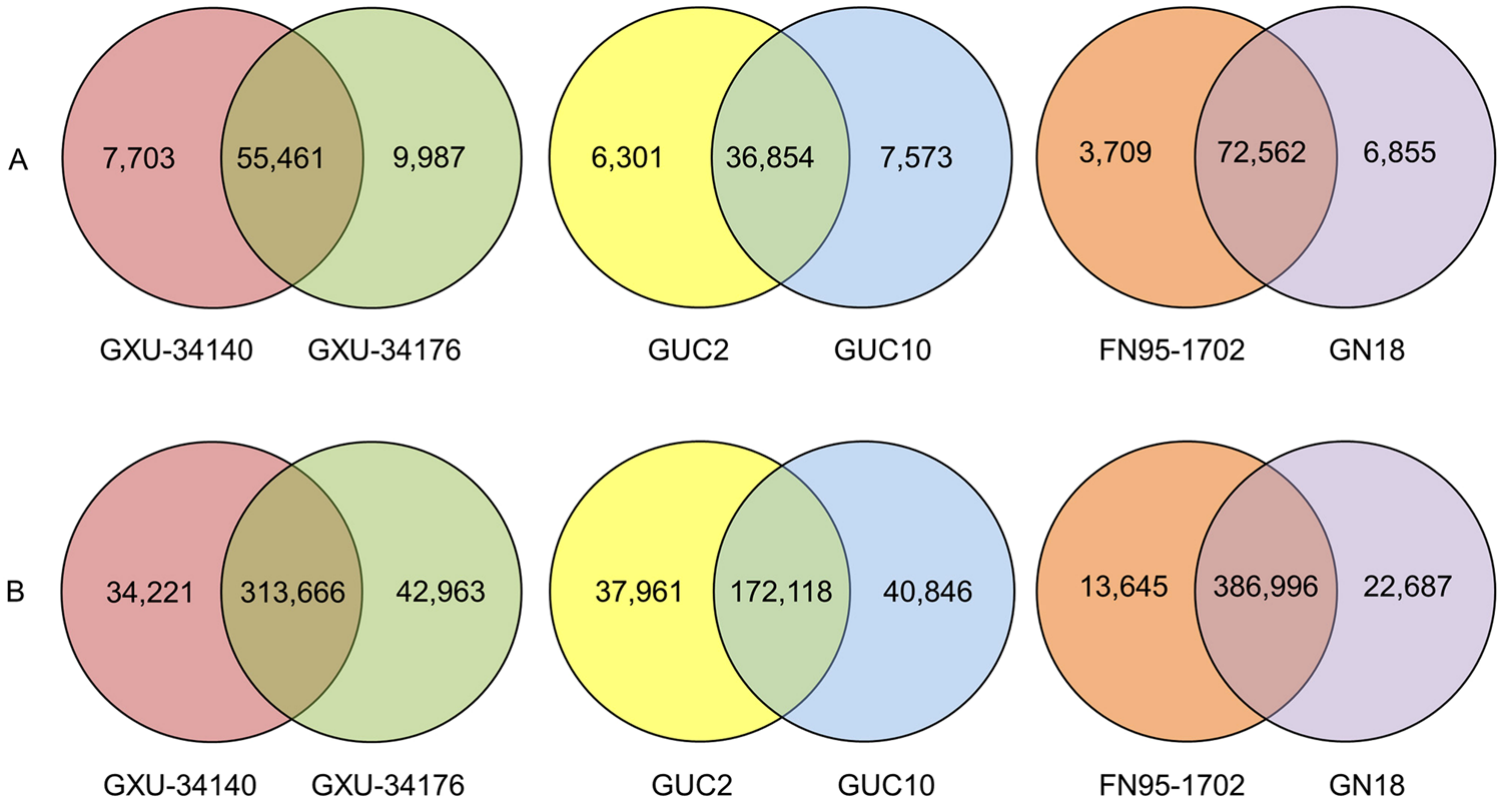
Venn diagram showing the number of unique and shared unigenes and SNPs among the contrasting sugarcane genotypes. (**A**) The number of unique and shared unigenes determined based on RSEM analysis. (**B**) The number of identified unique and shared putative SNPs based on GATK2 analysis. Only SNPs with distance >5 were retained.

Based on the RNA-seq data, the unique and shared unigenes were determined among the contrasting sugarcane cultivars (Fig. 4A). GXU-34140 and GXU-34176 shared a total of 55,461 (75.82%) unigenes, and GXU-34176 exhibited a higher number of unique unigenes (9,987) than those of GXU-34140 (7,703). A total of 72,562 (87.29%) unigenes were co-expressed in GN18 and FN95–1702 under drought stress, but the number of uniquely expressed unigenes in GN18 (6,855) was larger than that in FN95–1702 (3,709). GUC2 and GUC10 shared 36,854 (72.65%) unigenes, while the pokkah boeng disease susceptible GUC10 exhibited a higher number of unique unigenes (7,573) than the resistant cultivar GUC2 (6,301).

A total of 7,838 differentially expressed genes (DEGs) were identified between GXU-34140 and GXU-34176, with 4,582 up-regulated and 3,062 down-regulated in GXU-34176 (both +1 and +3 leaf sheath samples) when compared with GXU-34140. The remaining 194 DEGs showed the opposite expression pattern. According to GO functional enrichment analysis, GO terms, including ‘abscisic acid-activated signaling pathway’ (22 unigenes) and ‘jasmonic acid mediated signaling pathway’ (24 unigenes), were enriched in the up-regulated DEGs, while ‘cellulose synthase (UDP-forming) activity’ (26 unigenes), ‘cellulose biosynthetic process’ (30 unigenes), ‘plant-type primary cell wall biogenesis’ (7 unigenes), and ‘cell wall’ (56 unigenes) were enriched in the down-regulated DEGs (Supplementary Table S4). These results suggested that those genes might play an important role in responsive to leaf abscission for the easy defoliation GXU-34176.

In the comparison of GN18 with FN95–1702, a total of 5,314 DEGs were identified, of which 3,428 DEGs were up-regulated and 1,839 DEGs were down-regulated in GN18 responses to drought stress (both in mild drought and severe drought). The remaining 47 DEGs showed the opposite pattern. GO functional enrichment analysis indicated that ‘photosynthesis’ (23 unigenes), ‘chloroplast thylakoid membrane’ (36 unigenes), and ‘photosystem I’ (7 unigenes) were enriched in the up-regulated DEGs (Supplementary Table S4). These results suggested that interference of photosynthesis was less affected by drought stress in GN18 when compared with FN95–1702.

A total of 3,645 DEGs were identified in the comparison between GUC2 and GUC10, of which 2,175 DEGs were up-regulated and 1,454 DEGs were down-regulated in pokkah boeng resistant GUC2 (both in healthy and infected samples). Only 16 DEGs showed the opposite pattern. GO functional enrichment analysis indicated that ‘protein phosphorylation’ (162 unigenes), ‘protein serine/threonine kinase activity’ (139 unigenes), ‘transmembrane receptor protein serine/threonine kinase signaling pathway’ (16 unigenes), and ‘response to salicylic acid’ (19 unigenes) were enriched in the up-regulated DEGs (Supplementary Table S4), suggesting that they might represent special mechanisms in GUC2 responses to pokkah boeng disease.

### Quantitative Real-time PCR (qRT-PCR) validation of target genes

**Figure 5:**
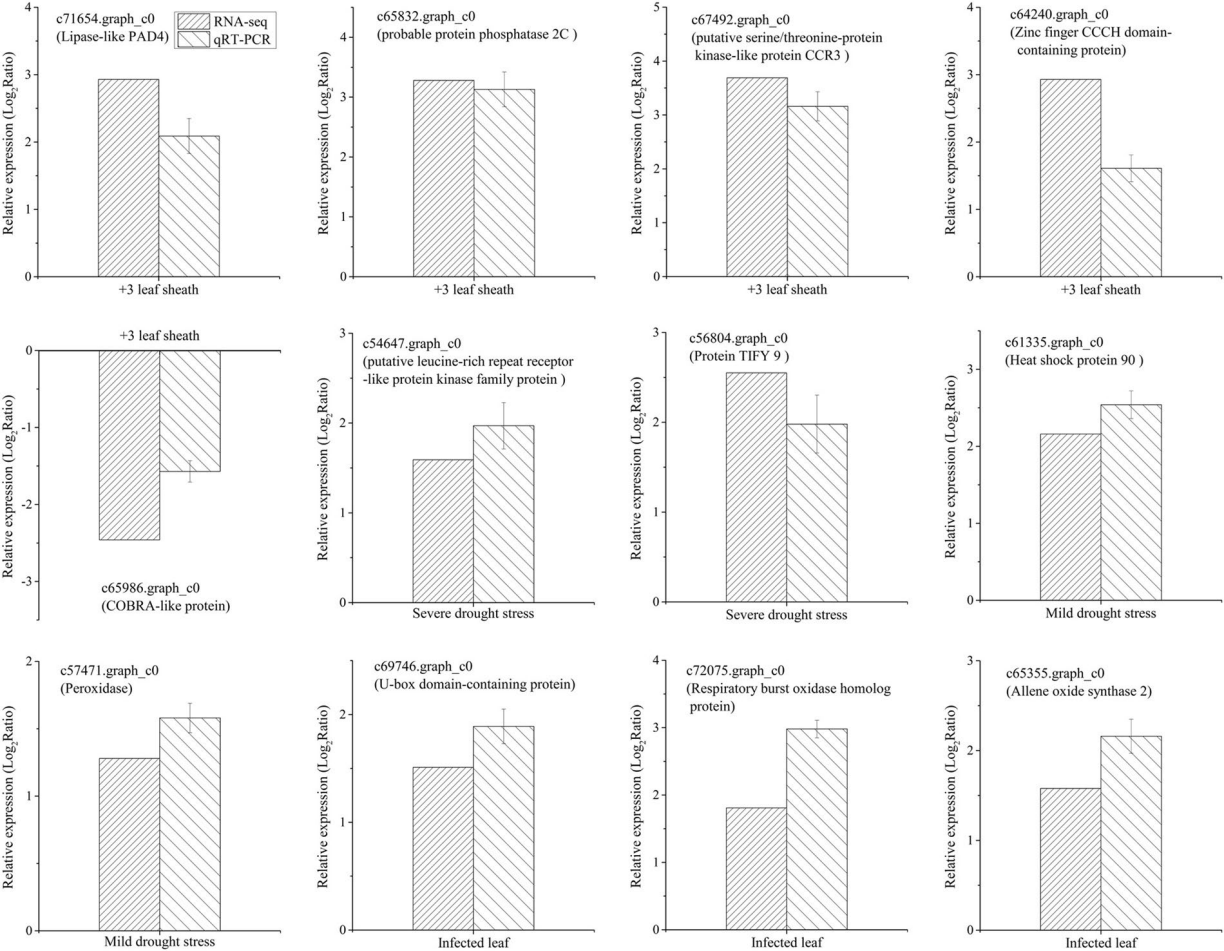
Verification of RNA-seq results by qRT-PCR. The DEGs in GXU-34176 vs GXU-34140 related to the category of ‘abscisic acid-activated signaling pathway’ (c71654.graph_c0 and c65832.graph_c0), ‘leaf senescence’ (c64240.graph_c0), ‘response to ethylene’ (c67492.graph_c0) and ‘cell wall modification involved in abscission’ (c65986.graph_c0). The DEGs in GN18 vs FN95–1702 related to the category of ‘response to water deprivation’ (c54647.graph_c0, c56804.graph_c0 and c61335.graph_c0) and ‘response to oxidative stress’ (c57471.graph_c0). The DEGs in GUC2 vs GUC10 related to the category of ‘ubiquitin-protein transferase activity’ (c69746.graph_c0), ‘defense response to fungus’ (c72075.graph_c0), ‘response to jasmonic acid’ (c65355.graph_c0). Data of qRT-PCR are presented as mean ± SD (n = 9) and error bars represent SD.

To validate the reliability and reproducibility of the Illumina RNA-Seq results, fourteen unigenes based on their characterizations of contrasting sugarcane genotypes were validated via qRT-PCR analysis. The primers used for qRT-PCR are listed in Supplementary Table S5. In GXU-34176, c71654.graph_c0 (encoding lipase-like PAD4) and c65832.graph_c0 (encoding probable protein phosphatase 2 C) related to ‘abscisic acid-activated signaling pathway’, c67492.graph_c0 (encoding putative serine/threonine-protein kinase-like protein CCR3) associated with ‘response to ethylene’ and c64240.graph_c0 (encoding zinc finger CCCH domain-containing protein) related to ‘leaf senescence’ were up-regulated in +3 leaf sheath compared to GXU-34140 (Fig. 5). Interestingly, the c65986.graph_c0 (encoding COBRA-like protein) related to ‘cell wall modification involved in abscission’ was down-regulated in +3 leaf sheath. In GN18, c54647.graph_c0 (encoding putative leucine-rich repeat receptor-like protein kinase family protein), c56804.graph_c0 (encoding protein TIFY 9), and c61335.graph_c0 (encoding heat shock protein 90) associated with ‘response to water deprivation’ as well as c57471.graph_c0 (encoding peroxidase) related to ‘response to oxidative stress’ were up-regulated under mild or severe drought stress compared to FN95–1702 (Fig. 5). In resistant cultivar GUC2, c69746.graph_c0 (encoding U-box domain-containing protein) associated with ‘ubiquitin-protein transferase activity’, c72075.graph_c0 (encoding respiratory burst oxidase homolog protein) related to ‘defense response to fungus’, and c65355.graph_c0 (encoding allene oxide synthase 2) related to ‘response to jasmonic acid’ were up-regulated after infected with *F*. *verticillioides* compared to sensitive cultivar GUC10 (Fig. 5). Pearson correlation coefficient between RNA-Seq and qRT-PCR was 0.89 and the Significance (two-tailed t test) was 1.11 × 10^−4^. These results showed that the expression trend of these genes was consistent with the transcriptome data.

### Putative marker discovery for marker-selection of important traits

**Figure 6:**
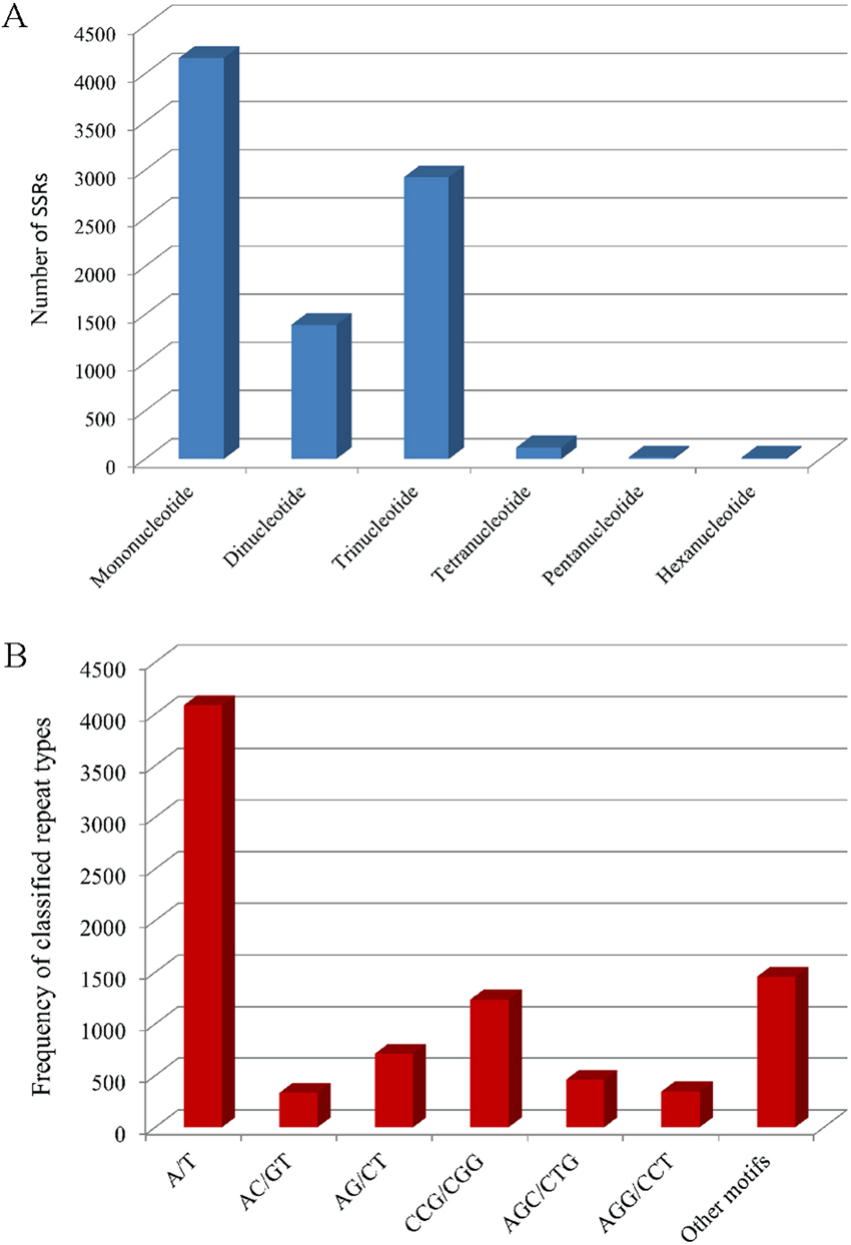
Characterization of potential SSR markers using MISA software. A total of 8,630 SSRs were mined from 6,883 unigenes. (**A**) Distribution of the different nucleotide repeat types. The most abundant repeat motifs were mononucleotide (4,162; 48.23%), followed by trinucleotide (2,924; 33.88%). (**B**) Frequency of the different classified repeat motifs. The repeat types of A/T and CCG/CGG accounted for 47.32% and 14.33%, respectively.

SSR markers are important tools for studying genetic diversity, constructing genetic maps, and performing comparative genomics^25^. Potential SSR markers were detected from unigenes with lengths over 1,000 bp using MISA software. A total of 8,630 SSRs were mined from 6,883 unigenes, of which 1,413 sequences contained more than one SSR and 286 SSRs were present in a compound formation (Supplementary Table S6). On average, the distribution density of SSRs was 1/6.62 kb. The most abundant repeat motifs were mononucleotide (4,162; 48.23%) and trinucleotide (2,924; 33.88%), followed by dinucleotide (1,392; 16.13%) and tetranucleotide (120; 1.39%) (Fig. 6A). Pentanucleotide and hexanucleotide repeat motifs represented only 0.22% and 0.15% of the total SSRs, respectively. The identified proportion of trinucleotide repeats were similar to the survey in the sugarcane EST (SUCEST) database (30.5%), but the percentage of tetranucleotides was lower than that obtained in the previous report^26^. Taken together, 188 types of nucleotide motif repeats were detected among 8,630 SSR loci. The most abundant repeat type was A/T (4,084; 47.32%), followed by CCG/CGG (1,237; 14.33%), AG/CT (712; 8.25%) and AGC/CTG (460; 5.33%) (Fig. 6B). These results were similar to those of the SSR motif previously reported^20^. Based on the 8,630 SSRs, primer pairs were designed using Primer 3.0 and are listed in Supplementary Table S6. These data are valuable resources for further studies on marker-assisted selection in sugarcane breeding.

SNPs are also widely used as molecular markers for the identification of quantitative trait locus, evolutionary analysis, and development marker-assisted selection to accelerate plant breeding. A total of 442,152 putative SNP positions were identified in 55,659 different unigenes (Supplementary Table S7). The unique and shared SNPs between the contrasting sugarcane genotypes were evaluated in Fig. 4B. The number of shared SNPs were 313,666 (80.25%) for GXU-34140 and GXU-34176, 172,118 (68.59%) for GUC2 and GUC10, and 386,996 (91.42%) for GN18 and FN95–1702, respectively. More shared SNPs resulted from the same parent of GXU-34140 and GXU-34176 or GN18 derived from FN95–1702 mediated with the *Ea-DREB2B* gene. A total of 77,184 SNPs (in 30,171 unigenes), 78,807 SNPs (in 25,029 unigenes), and 36,332 SNPs (in 19,719 unigenes) were different between GXU-34140 and GXU-34176, GUC2 and GUC10, and GN18 and FN95–1702, respectively. According to the GO annotation of unigenes that contained the unique SNPs in each group (Supplementary Table S8), several important categories were found to be associated with the sugarcane genotypes. For GXU-34140 and GXU-34176, 138 unigenes with SNPs were confirmed in the ‘response to abscisic acid’ category, 34 unigenes with SNPs in the ‘response to ethylene’ category, 17 unigenes with SNPs in the ‘leaf senescence’ category, and 17 unigenes with SNPs in the ‘cell wall modification’ category. Only one unigene with SNP was identified in the ‘cell wall modification involved in abscission’. For GUC2 and GUC10, 58 unigenes with SNPs were observed in the ‘defense response to fungus’, 17 in the ‘defense response to fungus, incompatible interaction’, 25 in the ‘response to fungus’, one in the ‘regulation of defense response to fungus, incompatible interaction’, and four in the ‘jasmonic acid and ethylene-dependent systemic resistance’. For GN18 and FN95–1702, 164 unigenes with SNPs were observed in the ‘defense response’, 50 in the ‘response to stress’, five in the ‘response to desiccation’, 49 in the ‘response to oxidative stress’, 45 in the ‘response to water deprivation’, and seven in the ‘water transport’. The SNPs derived from transcriptome sequences (in the transcribed regions) may be directly linked to expressed genes. The SNPs unique to each genotype are useful for developing markers associated with important agronomic traits of the contrasting genotypes.

## Discussion

Biotic and abiotic stresses in nature seriously restrict the development of the sugarcane industry worldwide. Currently the major aims of sugarcane breeding programs are to develop cultivars with strong resistance to phytopathogens (particularly *Ustilago scitaminea* and *Fusarium* species complex) and abiotic stress (especially drought stress), high sucrose and yield, and suitable for mechanized harvest. In our experiments, six sugarcane genotypes with contrasting responses to stress (drought, pokkah boeng disease) and defoliation were sequenced using the Illumina sequencing platform and 93,115 unigenes were assembled with an N50 of 1,774 bp, of which 43,526 unigenes were annotated against at least one of the public databases. The more assembled and annotated sequences in our studies indicated a comprehensive reference transcriptome of sugarcane when compared with other reports^20,21^. Many unigenes were identified in the signal transduction (1,333) and response to stimulus (5,937) categories, which were conducive to understanding the resistance mechanism.

Leaf abscission, which is one of the important traits for the sugarcane breeding program, is beneficial for improving the efficiency of sugarcane harvest. Two contrasting sugarcane genotypes (GXU-34140 with difficult-defoliation and GXU-34176 with easy-defoliation) derived from the same cross Co1001 × ROC22 showed different defoliation ability from our previous studies. ABA and ethylene played a more important role in the abscission of organs^27–29^. Compared to the leaf packaging sugarcane varieties (Q2 and B), ten transcripts involved in ‘abscisic acid associated pathways’ were up-regulated in leaf abscission sugarcane varieties (Q1 and T)^30^. In this work, more unigenes involved in ‘abscisic acid-activated signaling pathway’ (22 unigenes) and ‘response to abscisic acid’ (38 unigenes) were up-regulated in easy-defoliation GXU-34176 when compared to difficult-defoliation GXU-34140. Interestingly, 15 unigenes involved in ‘response to ethylene’ were up-regulated in GXU-34176, which was not observed in leaf abscission sugarcane varieties (Q1 and T)^30^. The position of organ separation from the plant body is called abscission zones (AZs). In *Phaseolus vulgaris petioles*, ethylene may induced the formation of AZs^31^. A total of 10 unigenes related to ‘cell wall macromolecule catabolic processes’ were up-regulated and 30 unigenes involved in ‘cellulose biosynthetic processes’ were down-regulated in GXU-34176. It is well-known that the common feature of abscission processes is cell wall degradation^32^. Genes involved in cell-wall modification during the abscission period have also been identified in Citrus and Tomato^33,34^.

Sugarcane pokkah boeng disease, caused by *Fusarium* species complex, is one of the most serious and devastating diseases recorded in countries where sugarcane is grown^35^. It has reportedly caused a yield loss of 40.8–64.5% in infection-susceptible sugarcane cultivars^36,37^. Based on our field survey and inoculation test, GUC2 was a promising genotype resistant to pokkah boeng disease, whereas GUC10 was susceptible to pokkah boeng. Comparative transcriptomic analysis indicated that 12 and 19 unigenes involved in response to jasmonic acid and salicylic acid were up-regulated in resistant GUC2. Salicylic acid (SA)-dependent signaling pathways and jasmonic acid (JA)-dependent signaling pathways are thought to form the backbone of the plant defense system^38^. In general, SA-mediated defenses are activated to resist biotrophic pathogens, whereas JA-mediated defenses confer resistance against necrotrophic pathogens that kill host cells^39,40^. SA and JA has been reported as defense elicitors to enhance resistance to Dutch elm disease in the tolerant phenotype of American elm^41^. The ubiquitin-proteasome system plays important roles in the regulation of plant immunity, especially the E3 ubiquitin ligases^42^. The results presented here showed one and 14 unigenes encoding an ubiquitin conjugating enzyme and ubiquitin-protein transferase activity were up-regulated in GUC2, respectively. In addition, our data also revealed that more than 100 unigenes encoding protein serine/threonine kinases were up-regulated in response to *Fusarium* species infection in GUC2. Future studies should focus on how pathogens and elicitors are perceived by protein serine/threonine kinases and how protein serine/threonine kinases and their activated signaling pathways are regulated during the *Fusarium* infection process in GUC2.

Natural disasters, such as drought and low temperature, occur frequently and have caused serious loss to sugarcane production^43^. Therefore, the development of sugarcane cultivars tolerant to abiotic stresses is crucial to improve the profitability of sugarcane industries. Transgenic technology can improve the efficiency of genetic improvement in many crops^44,45^. GN18 has improved drought resistance and was generated from FN95–1702 mediated with *Ea-DREB2B* gene^46^. The DREB transcription factors regulate the expression of many cold or/and drought-inducible genes in an ABA-independent pathway^47^. It has been reported that overexpression of *Ea-DREB2* in sugarcane leads to a higher photosynthetic rate and chlorophyll content compared to wild-type sugarcane during drought stress^48^. Comparative transcriptomic analyses revealed that unigenes encoding photosynthesis (23 unigenes), chloroplast thylakoid membrane (36 unigenes), and photosystem I (7 unigenes) were up-regulated in GN18. A total of 21 and four unigenes encoding peroxidase and antioxidant activity were up-regulated in GN18, respectively, indicating that GN18 had a high ability to scavenge reactive oxygen species (ROS)^49^. Several pathways involved in signal transduction were also up-regulated in GN18 in response to drought stress, including signal transduction (13 unigenes), signal transducer activity (five unigenes), kinase activity (26 unigenes), response to stress (14 unigenes), and response to water deprivation (10 unigenes).

Comparing transcriptomes of contrasting sugarcane genotypes was expected to identify a large collection of diverse unigenes that could support high-throughput computational identification of gene-associated SNPs. The results presented here showed that a total of 442,152 putative SNPs were detected in 55,659 unigenes, of which 77,184 SNPs in 30,171 unigenes, 78,807 SNPs in 25,029 unigenes, and 36,336 SNPs in 19,719 unigenes were different between GXU-34140 and GXU-34176, GUC2 and GUC10, and GN18 and FN95–1702, respectively. These unique SNPs in each contrasting genotype were associated with a different category in GO term annotation. For defoliation of GXU-34140 and GXU-34176, 138 unigenes with SNPs were confirmed in the ‘response to abscisic acid’ category, 34 in the ‘response to ethylene’, 17 in the ‘leaf senescence’, 17 in the ‘cell wall modification’ category, and only one in the ‘cell wall modification involved in abscission’. For pokkah boeng resistance of GUC2 and GUC10, 58 unigenes with SNPs were observed in the ‘defense response to fungus’, 17 in the ‘defense response to fungus, incompatible interaction’, 25 in the ‘response to fungus’, four in the ‘jasmonic acid and ethylene-dependent systemic resistance’, and only one in the ‘regulation of defense response to fungus, incompatible interaction’. For drought-tolerance of GN18 and FN95–1702, 164 unigenes with SNPs were observed in the ‘defense response’, 50 in the ‘response to stress’, 49 in the ‘response to oxidative stress’, 45 in the ‘response to water deprivation’, and seven in the ‘water transport’. These unique gene-based SNPs will be used to develop molecular markers associated with important agronomic characteristics of the contrasting genotypes.

In conclusion, sequences from six contrasting sugarcane genotypes were assembled and annotated by several public databases in order to obtain a comprehensive reference transcriptome of sugarcane. Comparative transcriptomic analysis revealed that the abscisic acid, ethylene, and the processes involved in cell-wall modification may play an important role in leaf abscission. The GN18 (transgenic lines mediated with *Ea-DREB2B* from FN95–1702) showed stronger drought tolerance with higher photosynthetic capacity and ROS scavenging capacity during drought stress. Many up-regulated DEGs related to signal transduction in GN18 may confer rapid adjustment the expression of stress related genes in response to drought stress. It also suggests that SA and JA as defense elicitors associate with other defense networks to build a mechanism to prevent pokkah boeng disease. Many gene-based SNPs have been identified as molecular markers to assist in improving sugarcane breeding programs. Taken together, the sequence and annotation resources will likely facilitate novel gene discovery and functional genomic studies in sugarcane even though a reference genome is currently lacking.

## Materials and Methods

### Plant Materials

Six contrasting sugarcane genotypes from Guangxi University (Nanning, China) were used for the transcriptome analysis based on their agronomic traits and abiotic and/or biotic resistance. The sugarcane of those genotypes was cut into single buds and sterilized with 0.3% carbendazim for 30 min. GXU-34140 with difficult defoliation and GXU-34176 with easy defoliation were derived from the same cross of ROC22 and Co1001 (see Supplementary Fig. 1A). Leaf sheath from GXU-34140 and GXU-34176 located at different leaf positions (+1, +3) were sampled during their mature period. GUC2 is a promising genotype resistant to pokkah boeng disease, whereas GUC10 is susceptible. Spore suspension of *F*. *verticillioides* prepared from 7-day-old colony on PDA medium and the spore concentration was adjusted to 1 × 10^6^ spores per ml using hemocytometer. At the elongating stage, the spore suspension containing 0.05% of Tween 20 was sprayed uniformly on leaves and kept high humidity and temperature for 3 days in the greenhouse. The sterile water containing 0.05% of Tween 20 was used as control. After 15 days, healthy (control) and infected leaves (the junction in health and disease) were collected and identified by PCR from GUC2 and GUC10 respectively (see Supplementary Fig. 1B,C)^50^. The GN18 and FN95–1702 were sown in plastic pot filled with the same weight of organic matter and field soil (1:1 in weight). The plot was kept in the glasshouse under natural conditions and watered daily to maintain soil moisture content close to filed capacity. Plants were grown under well-watered conditions until they showed three or four pieces leaves. Drought-tolerant GN18 and susceptible wild-type genotype (FN95–1702) were subjected to different levels of water deficit (well-watered, mild drought stress, severe drought stress and re-watered) by monitoring soil moisture content and membrane permeability during the seedling stage (see Supplementary Fig. 2)^46,51^. The leaf at +1 position was sampled from GN18 and FN95–1702. Totally, 48 samples (36 leaf samples and 12 leaf sheath samples) were collected from six contrasting genotypes exposed to biotic and abiotic stress as well as leaf defoliation. To obtain a good representation of sugarcane transcriptome, leave were sampled from 10 whole sugarcane plant (GN18 and FN95–1702) exposed to drought stress at different level of drought severity (well-watered, mild drought, severe drought) and re-watering, respectively. The symptomatic leaf sections were sampled from more than 20 inoculated sugarcane plants of GUC2 and GUC10, respectively. The leaf sheathes were taken at the first and the third visible dewlap from 10 plants of GXU-34140 and GXU-34176. Three biological replicates were collected and pooled for each sample from leaf and leaf sheath, and then immediately frozen in liquid nitrogen and stored at −80 °C until use. Therefore, for each contrasting genotype, more than 6 samples were used for transcriptome analysis.

### Library preparation for transcriptome sequencing

Total RNA was isolated and purified with Quick-RNATM Miniprep kit (Zymo Research, USA) according to the manufacturer’s instructions. The purity and integrity of RNA were assessed using a NanoPhotometer® spectrophotometer (IMPLEN, CA, USA). The RNA concentration was quantified using the Qubit® RNA Assay Kit on a Qubit®2.0 Fluorometer (Life Technologies, CA, USA). The RNA for sequencing library was pooled at same amount of each sample from same treatment. Sequencing libraries were constructed according to the manufacturer’s instructions using NEBNext®Ultra™ RNA Library Prep Kit for Illumina® (NEB, USA). The quality of the library was assessed on an Agilent Bioanalyzer 2100 system. Triplicate biological replicates were pooled and used for Illumina deep sequencing. Finally, sixteen cDNA libraries were sequenced on Illumina Hiseq. 2500 platform and paired-end reads were generated.

### Quality control and assembly

Before transcriptome assembly, the quality of raw reads was assessed by FastQC (http://www.bioinformatics.babraham.ac.uk/projects/fastqc/) and filtered using the NGS QC Toolkit to obtain high-quality clean reads^52^. First, reads containing adapters or poly-N as well as reads with more than 10% of bases with a poor quality score (Q < 20) were removed to generate high quality clean data. Then the sequencing reads mapped to *F*. *verticillioides* genome sequence were also removed. At the same time, Q20, GC-content, and the sequence duplication level of the clean data were calculated. All downstream analyses were based on clean data with high quality. *De novo* assembly was carried out on the cleaned reads using Trinity software by default^53^.

### Gene functional annotation

For functional annotation, all assembled unigenes were searched against the following databases: NR, COG, KOG, Swiss-Prot, KEGG, and GO using BLAST with a cutoff E-value of 10^−5^. KEGG analysis was carried out using KOBAS2.0 software^54^. The unigene sequences were also aligned to the Pfam database to predict possible functions using HMMER software with an E-value 10^−10^ as the threshold^55^.

### SSRs and SNPs detection and primer design

MISA software (http://pgrc.ipk-gatersleben.de/misa/misa.html) was used to identify SSR loci from unigenes greater than 1,000 bp. The nucleotide motifs were searched with a minimum of 5 repeat units. SSR primer pairs were designed by Primer 3 software (http://primer3.sourceforge.net/releases.php). The clean reads of each sample were mapped to the assembled reference transcriptome to generate the bam alignment files using picard-tools (v1.41) and samtools (v0.1.18). GATK2 software was used to perform SNP calling^56^. Raw vcffiles were filtered with GATK standard filter method and other parameters (cluster Window Size: 10; MQ0 >  = 4 and (MQ0/(1.0*DP)) > 0.1; QUAL < 10; QUAL < 30.0 or QD < 5.0 or HRun > 5), and only SNPs with distance >5 were retained.

### Gene expression analysis

The unigene abundance was normalized using Reads Per Kilobase of exon model per Million mapped reads (FPKM) with RSEM software (RNA-seq by Expectation Maximization, http://deweylab. biostat.wisc.edu/rsem/)^57^. Unigenes were defined as the unique or shared expressed transcripts among contrasting sugarcane genotypes based on the FPKM value (FPKM > 0). Differential expression analysis of two conditions/groups was performed using the DESeq R package (1.10.1). The resulting P values were adjusted using the Benjamini and Hochberg’s approach for controlling the false discovery rate (FDR). Genes with a threshold of FDR ≤ 0.05 and an absolute value of log_2_^Ratio^ ≥ 1 were assigned as differentially expressed. Gene Ontology functional enrichment analysis of the DEGs was performed using the GOseq R package-based Wallenius non-central hyper-geometric distribution^58^.

### qRT-PCR analysis

qRT-PCR was carried out to validate the reliability of differentially expressed genes in the LightCycler 480 thermocycler (Roche). PCR primers were designed (https://www.Idtdna.com/primerquest/Home/Index). Total RNA (1 μg) of each sample was converted into cDNA using the Transcriptor First Strand cDNA Synthesis Kit (Roche) according to the manufacturer’s instructions. The qRT-PCR reaction was performed in 20 μl containing 2 μl of template cDNA, 1.6 μl of primer mix (10 μm each of forward and reverse primers), 6.4 μl of sterile water, and 10 μl of 2 × SYBR Premix Ex TaqTMII (TaKaRa Bio Inc., Dalian). Amplifications were performed under the following conditions: 1 cycle of 30 s at 95 °C, followed by 45 cycles of 5 s at 95 °C and 30 s at 60 °C, with a final 30 s at 50 °C. The sugarcane housekeeping 25 S *rRNA* gene served as the internal reference gene to normalize gene expression level^59^. Three biological and three technical replications were performed for each sample. The data were analyzed using LightCycler® 480 sofware version 1.5.1 provided by Roche. The relative fold change of the selected genes was calculated using 2^−△△Ct^ algorithm^60^. The Pearson correlation test was performed by Origin 9.0 sofware.

## Electronic supplementary material

Supplementary Information

Supplementary Table S2

Supplementary Table S6

Supplementary Table S7

Supplementary Table S8
