# Supplementary material for: Transcriptomic characterization and potential marker development of contrasting sugarcane cultivars
Source: Sci Rep. 2018 Jan 26;8:1683. doi: 10.1038/s41598-018-19832-x (PMC5785991; doi:10.1038/s41598-018-19832-x)
Supplement: Supplementary file 1 — Supplementary Information [file 41598_2018_19832_MOESM1_ESM.pdf]

Supplementary Table S1-S8

Supplementary Figure S1-S2

Transcriptomic characterization and potential marker development of contrasting sugarcane cultivars

Shiqiang Xu[1#], Jihua Wang[1,2#], Heyang Shang[1], Youzong Huang[1], Wei Yao[1], Baoshan Chen[1], Muqing Zhang[1*]

[1]State Key Lab for Conservation and Utilization of Subtropical Agric-Biological Resources, Guangxi University, Nanning, 530005, China

[2] Crop Research Institute of Guangdong Academy of Agricultural Science, Guangzhou, 510640, China

[#] These authors contributed equally to this manuscripts.

*Corresponding author

mqzhang@ufl.edu

Supplementary Table S1. Summary of transcriptome sequencing data.

Genotypes	Samples	Clean reads	Clean bases	GC (%)	≥Q20(%)
GXU-34140	+1 leaf sheath	24,416,427	4,932,118,254	53.82	97.79
GXU-34140	+3 leaf sheath	25,980,099	5,247,979,998	53.46	97.83
GXU-34176	+1 leaf sheath	21,748,800	4,393,257,600	52.59	97.98
GXU-34176	+3 leaf sheath	23,500,334	4,747,067,468	53.59	98.14
GUC2	Healthy	28,984,065	5,854,781,130	56.39	97.95
GUC2	Infected	22,038,347	4,451,746,094	54.81	98.04
GUC10	Healthy	23,502,524	4,747,509,848	56.25	98.23
GUC10	Infected	21,126,025	4,267,457,050	55.18	98.24
GN18	Well-watered	31,954,528	6,454,814,656	52.06	99.05
GN18	Mild drought	34,237,151	6,915,904,502	53.55	98.97
GN18	Severe drought	34,176,917	6,903,737,234	51.29	99.02
GN18	Rewatered	36,928,627	7,459,582,654	52.29	99.05
FN95-1702	Well-watered	32,101,933	6,484,590,466	52.28	98.99
FN95-1702	Mild drought	35,759,273	7,223,373,146	54.15	98.93
FN95-1702	Severe drought	37,548,623	7,584,821,846	51.95	99.07
FN95-1702	Rewatered	31,506,687	6,364,350,774	53.39	98.96

Supplementary Table S3. Functional classification of sugarcane unigenes based on KEGG pathway.

Pathway	Pathway ID	Gene number
Glycolysis / Gluconeogenesis	ko00010	230
Citrate cycle (TCA cycle)	ko00020	120
Pentose phosphate pathway	ko00030	90
Pentose and glucuronate interconversions	ko00040	90
Fructose and mannose metabolism	ko00051	91
Galactose metabolism	ko00052	91
Ascorbate and aldarate metabolism	ko00053	66
Fatty acid biosynthesis	ko00061	60
Fatty acid elongation	ko00062	70
Fatty acid degradation	ko00071	91
Synthesis and degradation of ketone bodies	ko00072	15
Cutin, suberine and wax biosynthesis	ko00073	32
Steroid biosynthesis	ko00100	51
Ubiquinone and other terpenoid-quinone biosynthesis	ko00130	52
Oxidative phosphorylation	ko00190	327
Photosynthesis	ko00195	56
Photosynthesis-antenna proteins	ko00196	11
Purine metabolism	ko00230	228
Caffeine metabolism	ko00232	4
Pyrimidine metabolism	ko00240	169
Alanine, aspartate and glutamate metabolism	ko00250	103
Glycine, serine and threonine metabolism	ko00260	122
Cysteine and methionine metabolism	ko00270	143
Valine, leucine and isoleucine degradation	ko00280	96
Valine, leucine and isoleucine biosynthesis	ko00290	20
Lysine biosynthesis	ko00300	21
Lysine degradation	ko00310	73
Arginine and proline metabolism	ko00330	134
Histidine metabolism	ko00340	41
Tyrosine metabolism	ko00350	65
Phenylalanine metabolism	ko00360	176
Tryptophan metabolism	ko00380	68
Phenylalanine, tyrosine and tryptophan biosynthesis	ko00400	57
Benzoxazinoid biosynthesis	ko00402	2
beta-Alanine metabolism	ko00410	86
Taurine and hypotaurine metabolism	ko00430	19
Selenocompound metabolism	ko00450	29
Cyanoamino acid metabolism	ko00460	83
Glutathione metabolism	ko00480	177
Starch and sucrose metabolism	ko00500	233
N-Glycan biosynthesis	ko00510	66

Other glycan degradation	ko00511	29
Other types of O-glycan biosynthesis	ko00514	5
Amino sugar and nucleotide sugar metabolism	ko00520	166
Glycosaminoglycan degradation	ko00531	18
Glycerolipid metabolism	ko00561	111
Inositol phosphate metabolism	ko00562	92
Glycosylphosphatidylinositol(GPI)-anchor biosynthesis	ko00563	24
Glycerophospholipid metabolism	ko00564	150
Ether lipid metabolism	ko00565	50
Arachidonic acid metabolism	ko00590	24
Linoleic acid metabolism	ko00591	32
alpha-Linolenic acid metabolism	ko00592	67
Sphingolipid metabolism	ko00600	50
Glycosphingolipid biosynthesis-globo series	ko00603	16
Glycosphingolipid biosynthesis-ganglio series	ko00604	8
Pyruvate metabolism	ko00620	146
Glyoxylate and dicarboxylate metabolism	ko00630	118
Propanoate metabolism	ko00640	46
Butanoate metabolism	ko00650	41
C5-Branched dibasic acid metabolism	ko00660	6
One carbon pool by folate	ko00670	27
Carbon fixation in photosynthetic organisms	ko00710	136
Thiamine metabolism	ko00730	17
Riboflavin metabolism	ko00740	14
Vitamin B6 metabolism	ko00750	14
Nicotinate and nicotinamide metabolism	ko00760	17
Pantothenate and CoA biosynthesis	ko00770	32
Biotin metabolism	ko00780	23
Lipoic acid metabolism	ko00785	6
Folate biosynthesis	ko00790	20
Porphyrin and chlorophyll metabolism	ko00860	55
Terpenoid backbone biosynthesis	ko00900	70
Monoterpenoid biosynthesis	ko00902	18
Limonene and pinene degradation	ko00903	20
Diterpenoid biosynthesis	ko00904	25
Brassinosteroid biosynthesis	ko00905	20
Carotenoid biosynthesis	ko00906	40
Zeatin biosynthesis	ko00908	28
Sesquiterpenoid and triterpenoid biosynthesis	ko00909	6
Nitrogen metabolism	ko00910	60
Sulfur metabolism	ko00920	50
Phenylpropanoid biosynthesis	ko00940	249
Flavonoid biosynthesis	ko00941	49

Flavone and flavonol biosynthesis	ko00944	7
Stilbenoid, diarylheptanoid and gingerol biosynthesis	ko00945	33
Isoquinoline alkaloid biosynthesis	ko00950	32
Tropane, piperidine and pyridine alkaloid biosynthesis	ko00960	42
Glucosinolate biosynthesis	ko00966	1
Aminoacyl-tRNA biosynthesis	ko00970	105
Biosynthesis of unsaturated fatty acids	ko01040	81
Carbon metabolism	ko01200	452
2-Oxocarboxylic acid metabolism	ko01210	90
Fatty acid metabolism	ko01212	135
Degradation of aromatic compounds	ko01220	16
Biosynthesis of amino acids	ko01230	393
Vancomycin resistance	ko01502	2
ABC transporters	ko02010	71
Ribosome biogenesis in eukaryotes	ko03008	160
Ribosome	ko03010	1009
RNA transport	ko03013	294
mRNA surveillance pathway	ko03015	163
RNA degradation	ko03018	172
RNA polymerase	ko03020	58
Basal transcription factors	ko03022	60
DNA replication	ko03030	129
Spliceosome	ko03040	294
Proteasome	ko03050	122
Protein export	ko03060	78
Base excision repair	ko03410	59
Nucleotide excision repair	ko03420	134
Mismatch repair	ko03430	108
Homologous recombination	ko03440	126
Non-homologous end-joining	ko03450	11
Phosphatidylinositol signaling system	ko04070	96
Plant hormone signal transduction	ko04075	276
Ubiquitin mediated proteolysis	ko04120	189
Sulfur relay system	ko04122	11
SNARE interactions in vesicular transport	ko04130	39
Regulation of autophagy	ko04140	43
Protein processing in endoplasmic reticulum	ko04141	388
Endocytosis	ko04144	228
Phagosome	ko04145	156
Peroxisome	ko04146	137
Plant-pathogen interaction	ko04626	358
Circadian rhythm - plant	ko04712	50

Supplementary Table S4. GO functional enrichment of the DEGs between the contrasting sugarcane cultivars.

GO ID	GO term	Type	Number of DEGs	Corrected P-value
**The up-regulated DEGs in GXU-34176 compared to GXU-34140**				
GO:0004674	protein serine/threonine kinase activity	Molecular Function	282	0
GO:0009535	chloroplast thylakoid membrane	Cellular Component	70	0
GO:0010287	plastoglobule	Cellular Component	26	3.86E-11
GO:0010207	photosystem II assembly	Biological Process	34	1.54E-09
GO:0006468	protein phosphorylation	Biological Process	323	5.68E-08
GO:0010114	response to red light	Biological Process	22	4.2E-07
GO:0010200	response to chitin	Biological Process	29	4.31E-07
GO:0009657	plastid organization	Biological Process	17	1.5E-06
GO:0030247	polysaccharide binding	Molecular Function	41	2.29E-06
GO:0009773	photosynthetic electron transport in photosystem I	Biological Process	17	2.61E-06
GO:0006612	protein targeting to membrane	Biological Process	28	2.81E-06
GO:0010363	regulation of plant-type hypersensitive response	Biological Process	28	4.87E-06
GO:0043900	regulation of multi-organism process	Biological Process	15	8.39E-06
GO:0009637	response to blue light	Biological Process	19	1.15E-05
GO:0035304	regulation of protein dephosphorylation	Biological Process	21	1.3E-05
GO:0010218	response to far red light	Biological Process	20	1.83E-05
GO:0009941	chloroplast envelope	Cellular Component	73	2.83E-05
GO:0000165	MAPK cascade	Biological Process	25	3.07E-05
GO:0016168	chlorophyll binding	Molecular Function	11	3.34E-05
GO:0009862	systemic acquired resistance, salicylic acid mediated signaling pathway	Biological Process	21	3.62E-05
GO:0009595	detection of biotic stimulus	Biological Process	15	4.32E-05
GO:0009867	jasmonic acid mediated signaling pathway	Biological Process	24	6.22E-05
GO:0005509	calcium ion binding	Molecular Function	72	6.87E-05
GO:0070838	divalent metal ion transport	Biological Process	13	0.000254
GO:0009738	abscisic acid-activated signaling pathway	Biological Process	22	0.000554
GO:0009538	photosystem I reaction center	Cellular Component	5	0.000863
GO:0030003	cellular cation homeostasis	Biological Process	13	0.001081
GO:0009697	salicylic acid biosynthetic process	Biological Process	18	0.001915
GO:0048046	apoplast	Cellular Component	59	0.002852
GO:0031348	negative regulation of defense response	Biological Process	21	0.002885
GO:0009409	response to cold	Biological Process	49	0.003022
GO:0003700	sequence-specific DNA binding transcription factor activity	Molecular Function	102	0.003208
GO:0009522	photosystem I	Cellular Component	7	0.007117
GO:0010310	regulation of hydrogen peroxide metabolic process	Biological Process	16	0.007972
GO:0080167	response to karrikin	Biological Process	31	0.009561
GO:0019344	cysteine biosynthetic process	Biological Process	21	0.010437
GO:0031012	extracellular matrix	Cellular Component	6	0.011677
GO:0050832	defense response to fungus	Biological Process	25	0.012262
GO:0005986	sucrose biosynthetic process	Biological Process	8	0.015268
GO:0043085	positive regulation of catalytic activity	Biological Process	19	0.017546
GO:0009611	response to wounding	Biological Process	36	0.026824
GO:0030145	manganese ion binding	Molecular Function	12	0.027408
GO:0009765	photosynthesis, light harvesting	Biological Process	8	0.028112
GO:0006098	pentose-phosphate shunt	Biological Process	31	0.02812
GO:0031977	thylakoid lumen	Cellular Component	8	0.02812
GO:0004672	protein kinase activity	Molecular Function	77	0.028748

GO:0043069	negative regulation of programmed cell death	Biological Process	17	0.03598
GO:0009718	anthocyanin-containing compound biosynthetic process	Biological Process	9	0.040986
GO:0009523	photosystem II	Cellular Component	6	0.043968
GO:0004364	glutathione transferase activity	Molecular Function	15	0.045206
**The down-regulated DEGs in GXU-34176 compared to GXU-34140**				
GO:0016760	cellulose synthase (UDP-forming) activity	Molecular Function	26	2.59E-10
GO:0030244	cellulose biosynthetic process	Biological Process	30	8.04E-08
GO:0016021	integral component of membrane	Cellular Component	230	1.55E-06
GO:0020037	heme binding	Molecular Function	70	3.83E-06
GO:0043086	negative regulation of catalytic activity	Biological Process	24	1.35E-05
GO:0005506	iron ion binding	Molecular Function	68	2.28E-05
GO:0005576	extracellular region	Cellular Component	36	4.18E-05
GO:0005618	cell wall	Cellular Component	56	4.69E-05
GO:0000139	Golgi membrane	Cellular Component	20	5.11E-05
GO:0008017	microtubule binding	Molecular Function	25	6.09E-05
GO:0009538	photosystem I reaction center	Cellular Component	5	0.00018
GO:0016168	chlorophyll binding	Molecular Function	9	0.000191
GO:0055114	oxidation-reduction process	Biological Process	151	0.000302
GO:0005874	microtubule	Cellular Component	26	0.000356
GO:0004601	peroxidase activity	Molecular Function	32	0.000596
GO:0008289	lipid binding	Molecular Function	11	0.000663
GO:0007018	microtubule-based movement	Biological Process	21	0.00081
GO:0009522	photosystem I	Cellular Component	7	0.000922
GO:0005975	carbohydrate metabolic process	Biological Process	45	0.001144
GO:0009833	plant-type primary cell wall biogenesis	Biological Process	7	0.00179
GO:0004497	monooxygenase activity	Molecular Function	41	0.001824
GO:0005871	kinesin complex	Cellular Component	19	0.002169
GO:0005794	Golgi apparatus	Cellular Component	48	0.002377
GO:0016757	transferase activity, transferring glycosyl groups	Molecular Function	27	0.002779
GO:0009505	plant-type cell wall	Cellular Component	33	0.004877
GO:0030001	metal ion transport	Biological Process	15	0.005395
GO:0008146	sulfotransferase activity	Molecular Function	9	0.011025
GO:0018298	protein-chromophore linkage	Biological Process	8	0.011799
GO:0016705	oxidoreductase activity, acting on paired donors, with incorporation or reduction of molecular oxygen	Molecular Function	39	0.012551
GO:0046658	anchored component of plasma membrane	Cellular Component	13	0.016402
GO:0008422	beta-glucosidase activity	Molecular Function	7	0.019156
GO:0016747	transferase activity, transferring acyl groups other than amino-acyl groups	Molecular Function	26	0.022585
GO:0016165	linoleate 13S-lipoxygenase activity	Molecular Function	8	0.031727
GO:0006629	lipid metabolic process	Biological Process	33	0.033195
GO:0042802	identical protein binding	Molecular Function	20	0.033735
GO:0006979	response to oxidative stress	Biological Process	30	0.034339
GO:0006857	oligopeptide transport	Biological Process	14	0.043435
GO:0004553	hydrolase activity, hydrolyzing O-glycosyl compounds	Molecular Function	27	0.045165
**The up-regulated DEGs in GN18 compared to FN95-1702**				
GO:0015979	photosynthesis	Biological Process	23	0.000265
GO:0016556	mRNA modification	Biological Process	19	0.000527
GO:0016165	linoleate 13S-lipoxygenase activity	Molecular Function	10	0.000952
GO:0016760	cellulose synthase (UDP-forming) activity	Molecular Function	18	0.001176
GO:0009522	photosystem I	Cellular Component	7	0.00215
GO:0009833	plant-type primary cell wall biogenesis	Biological Process	7	0.003811

GO:0016168	chlorophyll binding	Molecular Function	8	0.006016
GO:0009535	chloroplast thylakoid membrane	Cellular Component	36	0.010543
GO:0006364	rRNA processing	Biological Process	30	0.039655
GO:0009765	photosynthesis, light harvesting	Biological Process	7	0.043179
GO:0019252	starch biosynthetic process	Biological Process	23	0.048773
GO:0010218	response to far red light	Biological Process	13	0.049614
**The down-regulated DEGs in GN18 compared to FN95-1702**				
GO:0003700	sequence-specific DNA binding transcription factor activity	Molecular Function	60	3.12E-07
GO:0006355	regulation of transcription, DNA-templated	Biological Process	90	3.9E-07
GO:0015996	chlorophyll catabolic process	Biological Process	14	4.5E-06
GO:0008152	metabolic process	Biological Process	57	0.000674
GO:0009535	chloroplast thylakoid membrane	Cellular Component	25	0.000706
GO:0055114	oxidation-reduction process	Biological Process	95	0.00106
GO:0000976	transcription regulatory region sequence-specific DNA binding	Molecular Function	7	0.002753
GO:0009750	response to fructose	Biological Process	11	0.004978
GO:0043565	sequence-specific DNA binding	Molecular Function	30	0.009669
GO:0004867	serine-type endopeptidase inhibitor activity	Molecular Function	8	0.011203
GO:0047274	galactinol-sucrose galactosyltransferase activity	Molecular Function	4	0.019844
GO:0080044	quercetin 7-O-glucosyltransferase activity	Molecular Function	7	0.020364
GO:0006950	response to stress	Biological Process	24	0.020784
GO:0004722	protein serine/threonine phosphatase activity	Molecular Function	12	0.027973
GO:0009788	negative regulation of abscisic acid-activated signaling pathway	Biological Process	7	0.028371
GO:0010319	stromule	Cellular Component	7	0.032873
**The up-regulated DEGs in GUC2 compared to GUC10**				
GO:0052716	hydroquinone	Molecular Function	12	1.35E-06
GO:0046274	lignin catabolic process	Biological Process	12	3.43E-06
GO:0004674	protein serine/threonine kinase activity	Molecular Function	139	9.21E-06
GO:0071805	potassium ion transmembrane transport	Biological Process	15	3.13E-05
GO:0006468	protein phosphorylation	Biological Process	162	3.49E-05
GO:0016021	integral component of membrane	Cellular Component	185	4.4E-05
GO:0004675	transmembrane receptor protein serine/ threonine kinase activity	Molecular Function	16	0.000601
GO:0015079	potassium ion transmembrane transporter activity	Molecular Function	9	0.00115
GO:0007178	transmembrane receptor protein serine/ threonine kinase signaling pathway	Biological Process	16	0.00162
GO:0005840	ribosome	Cellular Component	63	0.002307
GO:0009751	response to salicylic acid	Biological Process	19	0.002328
GO:0050664	oxidoreductase activity, acting on NAD(P)H, oxygen as acceptor	Molecular Function	6	0.002487
GO:0005887	integral component of plasma membrane	Cellular Component	8	0.008066
GO:0015798	myo-inositol transport	Biological Process	5	0.013496
GO:0045548	phenylalanine ammonia-lyase activity	Molecular Function	5	0.014458
GO:0009800	cinnamic acid biosynthetic process	Biological Process	5	0.029079
GO:0055085	transmembrane transport	Biological Process	55	0.033406
GO:0015576	sorbitol transmembrane transporter activity	Molecular Function	4	0.03813
GO:0015168	glycerol transmembrane transporter activity	Molecular Function	4	0.03813
GO:0015591	D-ribose transmembrane transporter activity	Molecular Function	4	0.03813
GO:0005365	myo-inositol transmembrane transporter activity	Molecular Function	4	0.03813
GO:0015148	D-xylose transmembrane transporter activity	Molecular Function	4	0.03813
GO:0015575	mannitol transmembrane transporter activity	Molecular Function	4	0.03813
GO:0043090	amino acid import	Biological Process	9	0.038919

GO:0010119	regulation of stomatal movement	Biological Process	11	0.041448
GO:0005507	copper ion binding	Molecular Function	28	0.041718
GO:0003735	structural constituent of ribosome	Molecular Function	72	0.042452
**The down-regulated DEGs in GUC2 compared to GUC10**				
GO:0009535	chloroplast thylakoid membrane	Cellular Component	80	0
GO:0009570	chloroplast stroma	Cellular Component	73	0
GO:0010207	photosystem II assembly	Biological Process	41	0
GO:0009657	plastid organization	Biological Process	16	0
GO:0009773	photosynthetic electron transport in photosystem I	Biological Process	19	2.75E-16
GO:0016168	chlorophyll binding	Molecular Function	12	4.77E-12
GO:0010287	plastoglobule	Cellular Component	18	1.37E-10
GO:0035304	regulation of protein dephosphorylation	Biological Process	20	1.4E-10
GO:0009637	response to blue light	Biological Process	17	1.41E-10
GO:0016117	carotenoid biosynthetic process	Biological Process	20	1.76E-10
GO:0010598	NAD(P)H dehydrogenase complex (plastoquinone)	Cellular Component	9	1.96E-10
GO:0015995	chlorophyll biosynthetic process	Biological Process	24	2.16E-10
GO:0043085	positive regulation of catalytic activity	Biological Process	20	3.15E-10
GO:0019344	cysteine biosynthetic process	Biological Process	21	3.16E-10
GO:0009941	chloroplast envelope	Cellular Component	84	3.33E-10
GO:0006098	pentose-phosphate shunt	Biological Process	48	5.91E-10
GO:0019288	isopentenyl diphosphate biosynthetic process, methylerythritol 4-phosphate pathway	Biological Process	40	5.92E-10
GO:0006364	rRNA processing	Biological Process	50	7.78E-10
GO:0010027	thylakoid membrane organization	Biological Process	28	8.51E-10
GO:0010218	response to far red light	Biological Process	17	8.88E-10
GO:0010114	response to red light	Biological Process	17	8.88E-10
GO:0070838	divalent metal ion transport	Biological Process	13	9.86E-10
GO:0019252	starch biosynthetic process	Biological Process	26	1.3E-09
GO:0009543	chloroplast thylakoid lumen	Cellular Component	16	1.44E-09
GO:0000023	maltose metabolic process	Biological Process	21	4.37E-09
GO:0030003	cellular cation homeostasis	Biological Process	13	5.08E-09
GO:0009522	photosystem I	Cellular Component	8	5.91E-07
GO:0009507	chloroplast	Cellular Component	80	1.84E-06
GO:0015979	photosynthesis	Biological Process	18	5.36E-06
GO:0009902	chloroplast relocation	Biological Process	16	6.75E-06
GO:0009538	photosystem I reaction center	Cellular Component	5	1.04E-05
GO:0010155	regulation of proton transport	Biological Process	11	2.06E-05
GO:0003700	sequence-specific DNA binding transcription factor activity	Molecular Function	51	3.28E-05
GO:0009765	photosynthesis, light harvesting	Biological Process	7	0.000283
GO:0009523	photosystem II	Cellular Component	6	0.000295
GO:0004364	glutathione transferase activity	Molecular Function	11	0.000338
GO:0009658	chloroplast organization	Biological Process	14	0.000438
GO:0006457	protein folding	Biological Process	28	0.000633
GO:0009534	chloroplast thylakoid	Cellular Component	10	0.000727
GO:0019761	glucosinolate biosynthetic process	Biological Process	13	0.000797
GO:0010264	myo-inositol hexakisphosphate biosynthetic process	Biological Process	9	0.00088
GO:0005528	FK506 binding	Molecular Function	8	0.001067
GO:0009579	thylakoid	Cellular Component	11	0.001505
GO:0009644	response to high light intensity	Biological Process	15	0.001773
GO:0042793	transcription from plastid promoter	Biological Process	11	0.002117
GO:0016629	12-oxophytodienoate reductase activity	Molecular Function	5	0.002489

GO:0015035	protein disulfide oxidoreductase activity	Molecular Function	12	0.005605
GO:0016491	oxidoreductase activity	Molecular Function	32	0.005969
GO:0046686	response to cadmium ion	Biological Process	36	0.006147
GO:0030093	chloroplast photosystem I	Cellular Component	3	0.007342
GO:0048046	apoplast	Cellular Component	31	0.008082
GO:0030076	light-harvesting complex	Cellular Component	4	0.008817
GO:0006355	regulation of transcription, DNA-templated	Biological Process	72	0.009552
GO:0003755	peptidyl-prolyl cis-trans isomerase activity	Molecular Function	11	0.011443
GO:0047100	glyceraldehyde-3-phosphate dehydrogenase (NADP+) (phosphorylating) activity	Molecular Function	3	0.011625
GO:0008289	lipid binding	Molecular Function	7	0.014798
GO:0009295	nucleoid	Cellular Component	5	0.015606
GO:0009508	plastid chromosome	Cellular Component	5	0.015606
GO:0019253	reductive pentose-phosphate cycle	Biological Process	5	0.018895
GO:0031072	heat shock protein binding	Molecular Function	10	0.023384
GO:0042549	photosystem II stabilization	Biological Process	3	0.025993
GO:0010258	NADH dehydrogenase complex (plastoquinone) assembly	Biological Process	3	0.025993
GO:0019684	photosynthesis, light reaction	Biological Process	7	0.026397
GO:0016556	mRNA modification	Biological Process	11	0.03077
GO:0009220	pyrimidine ribonucleotide biosynthetic process	Biological Process	12	0.036063
GO:0006636	unsaturated fatty acid biosynthetic process	Biological Process	8	0.0384
GO:0010103	stomatal complex morphogenesis	Biological Process	11	0.039328
GO:0016655	oxidoreductase activity, acting on NAD(P)H, quinone or similar compound as acceptor	Molecular Function	4	0.040224
GO:0009409	response to cold	Biological Process	23	0.041431
GO:0031408	oxylipin biosynthetic process	Biological Process	9	0.042043
GO:0010304	PSII associated light-harvesting complex II catabolic process	Biological Process	5	0.04516
GO:0045156	electron transporter, transferring electrons within the cyclic electron transport pathway of photosynthesis activity	Molecular Function	3	0.045413
GO:0000234	phosphoethanolamine N-methyltransferase activity	Molecular Function	3	0.045413

Supplementary Table S5. Primer information for the qRT-PCR analysis.

Gene ID	Forward (5'-3')	Reverse (5'-3')	Function
c71654.graph_c0	CTCAAGCTCAGGGATGAAGTG	GAATGCCCATCAGCTACCTAC	Lipase-like PAD4
c65832.graph_c0	AGGCTGAAGCAAACGGATAA	CTTGTCGTCTCATGTCGTCTC	probable protein phosphatase 2C
c64240.graph_c0	CGAGAGAAGTGACGGGTTTAC	CGCCATTCCATGCTCAAATC	Zinc finger CCCH domain-containing protein
c67492.graph_c0	TCGTACAGGGTGGTGGT	CTGCCGAGCGACCTATTC	putative serine/threonine-protein kinase-like protein CCR3
c65986.graph_c0	ATCTGTCGCCGTCTTCAATC	TTTCCCTCTGACGAAGCAATTA	COBRA-like protein
c69746.graph_c0	GGGCTCTCTTCATCAAGTTCTC	GATCACCAAGCTCTGCAGTTA	U-box domain-containing protein
c72075.graph_c0	CTATCGAAGATCACGGAGCAAG	CAGGTTGTAGAGCTCGATGTAG	Respiratory burst oxidase homolog protein
c65355.graph_c0	AGCGTCATTGGTTGGATATGA	CACGTGAAGAGGAAAGAGAGAG	Allene oxide synthase 2
c54647.graph_c0	CTTCCTATGTACACTGGCTACAG	GGTAGGAGTTCAGGTTGACTATG	putative leucine-rich repeat receptor-like protein kinase family protein
c56804.graph_c0	CTTGTGAGTGGCATGTCTTTG	GATGGCGATGGAGGTTACTAC	Protein TIFY 9
c61335.graph_c0	GGTCCGCAAACTCGTACTTATC	GTGGCTGACAGTAGCTCTTATG	Heat shock protein 90
c57471.graph_c0	GCAGCTGTCCTTGTAGTAGTC	CATGGAGAAGCGACGAAGAG	Peroxidase
25S rRNA	GCAGCCAAGCGTTCATAGC	CCTATTGGTGGGTGAACAATCC	The internal reference gene

Supplementary Table S2. Overview of the annotation of assembled unigenes.

Supplementary Table S6. Identified putative SSRs results.

Supplementary Table S7. The putative SNPs identified in this study.

Supplementary Table S8. The GO annotation of unigenes that contain the unique SNPs between the contrasting sugarcane cultivars.

(supplied as a separate file)

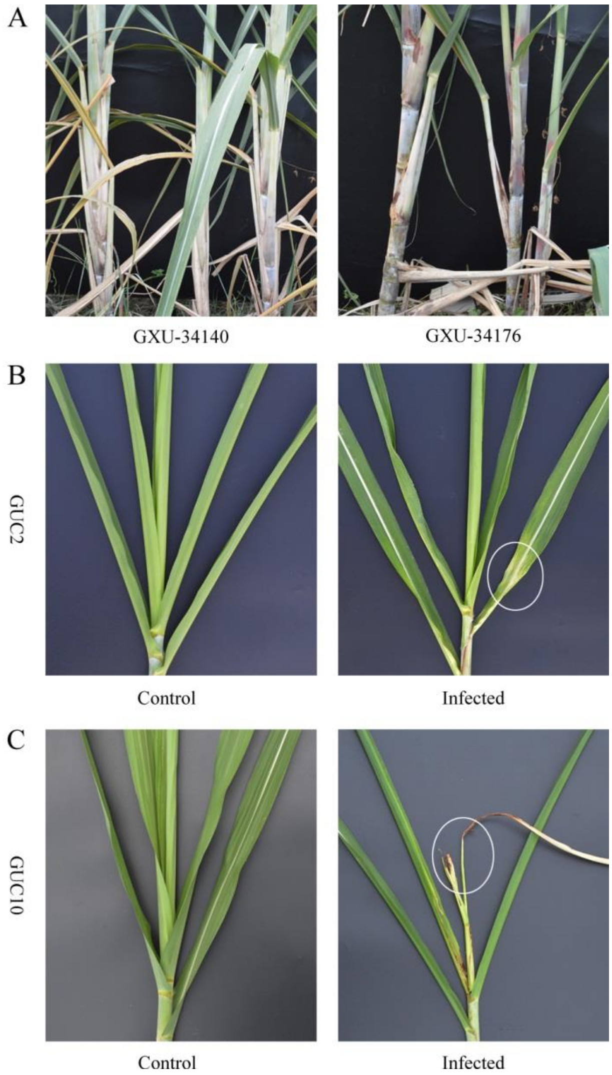

Supplementary Figure S1. Character of the resistant to pokkah boeng disease and defoliation. (A) The difficult defoliation genotype GXU-34140, which was wrapped tightly by leaves; The easy defoliation genotype GXU-34176, which can naturally shed leaves; (B) Resistant genotype GUC2, only the leaves becomeing slightly chlorotic and twisted after inoculation of *F. verticillioides*, which can resume growth sonn. (C) Susceptible genotype GUC10, the growing point of plant was rot after inoculation of *F. verticillioides*, which results in the die of the entire top of the plant.

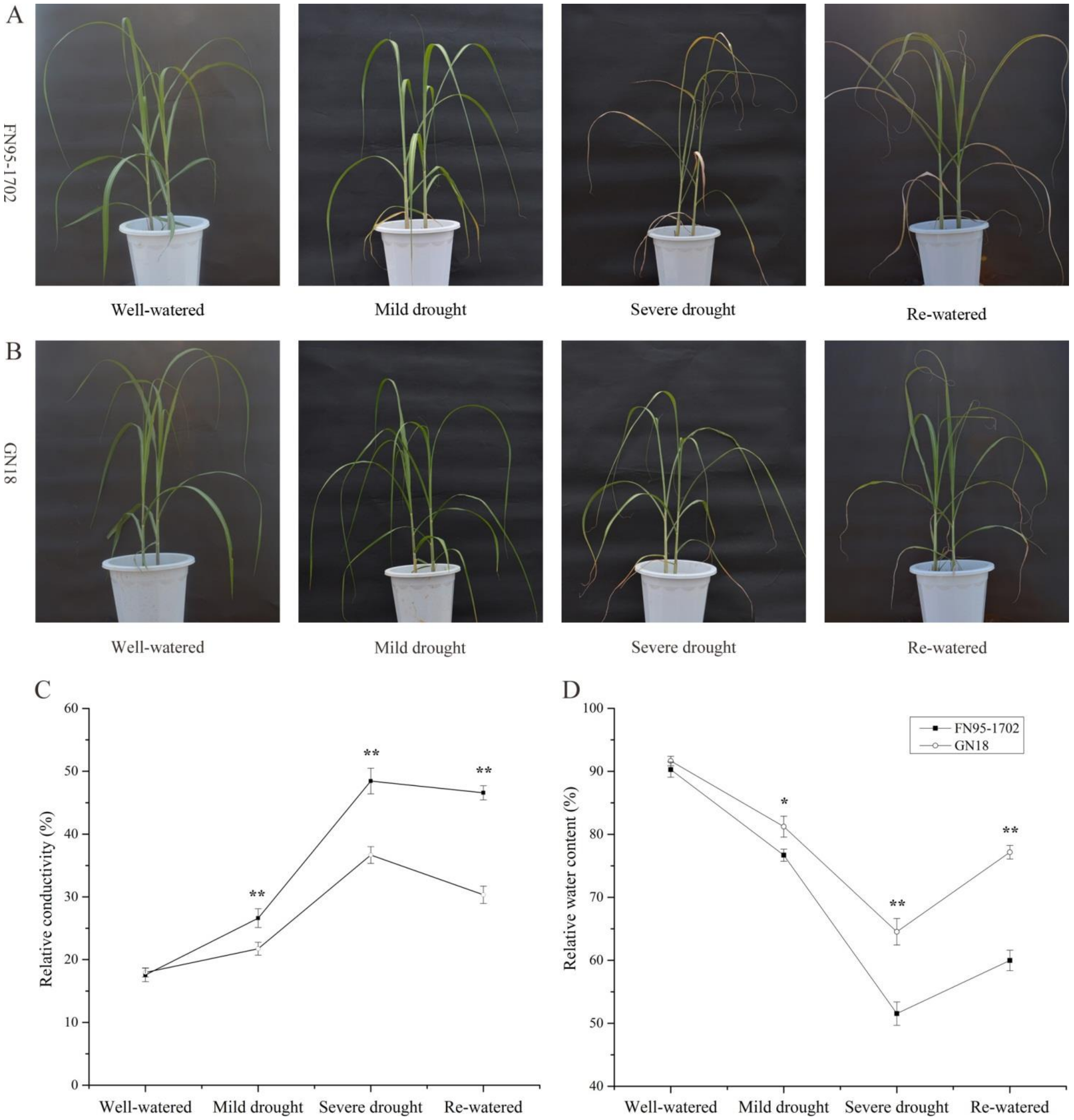

Supplementary Figure S2. Water deficit treament of FN95-1702 and GN18. (A) and (B), Well-watered and drought-stressed plants by withholding water and upon rewatering; (C) and (D), Effects of drought on leaf relative water content and relative conductivity. A, the plants of FN95-1702; B, the plants of GN18; Treament of re-waterwd represent 5-day after rewatering. Error bars represent SD (n=3 plants). Asterisks indicate significant differences that were observed (Student's t-test: *P<0.05; **P<0.01).
